# Differential physical activity responses to diabetes risk reduction interventions according to prediabetes phenotype in at-risk individuals: pooled analysis from three randomised controlled trials

**DOI:** 10.1007/s00125-026-06873-5

**Published:** 2026-09-14

**Authors:** Emily James, Joseph Henson, Melanie J. Davies, Kamlesh Khunti, Thomas Yates

**Affiliations:** 1 National Institute for Health and Care Research Leicester Biomedical Research Centre, Leicester, UK; 2 Diabetes Research Centre, College of Life Sciences, University of Leicester, Leicester, UK; 3 National Institute for Health and Care Research Applied Research Collaborations East Midlands, University Hospitals of Leicester NHS Trust, Leicester, UK

**Keywords:** Impaired fasting glucose, Impaired glucose tolerance, Lifestyle, Physical activity, Prediabetes, Prevention

## Abstract

**Aims/hypothesis:**

Emerging research suggests that the effects of lifestyle interventions to reduce the risk of type 2 diabetes progression differ by prediabetes phenotype. We investigated whether this divergent effectiveness is accompanied by differing physical activity responses to the intervention.

**Methods:**

Pooled data from three randomised controlled trials were used (*n*=2150), with up to 48 months annual follow-up. Participants at risk of type 2 diabetes were randomised to either: (1) usual care (control) or (2) similar interventions, in which they received structured group-based lifestyle interventions to promote increases in physical activity and other behaviours associated with diabetes risk reduction, supported by education, goal setting, pedometer use and maintenance support. Change in physical activity and glucose levels were stratified by the presence of impaired fasting glucose (IFG) and/or impaired glucose tolerance (IGT).

**Results:**

Participants in the group with normoglycaemia were 63 years old (mean), with a mean BMI of 29.9 kg/m^2^; 56.4% were men and 76.7% were White European. Participants in the IFG group were 63 years old, with a BMI of 29.8 kg/m^2^; 70.0% were men and 79.1% were White European. Participants in the IGT group were 65 years old, with a BMI of 31.1 kg/m^2^; 60.4% were men and 80.5% were White European. Participants in the group with both IFG and IGT were 65 years old, with a BMI of 31.7 kg/m^2^; 62.7% were men and 82.6% were White European. The number of baseline steps/day was 6130 ± 3201 in the normoglycaemia group, 7244 ± 3489 in the IFG group, 5984 ± 2904 in the IGT group and 6310 ± 2872 in the IFG+IGT group (means ± SD). The intervention increased the mean number of steps/day relative to usual care by 247 (95% CI 4, 491) in the normoglycaemia group, 523 (95% CI 229, 818) in the IGT group, and 808 (95% CI 402, 1213) in the IFG+IGT group, but not in the IFG group (mean 71; 95% CI −429, 571). The intervention improved 2 h post-challenge glucose levels in the IFG+IGT group only (mean −0.5 mmol/l; 95% CI −0.9, 0.0).

**Conclusions/interpretation:**

The effectiveness of structured, group-based interventions with goal setting to promote physical activity differs by glycaemic status; those with IFG were least responsive. This may contribute to previously observed differences in the clinical effectiveness of diabetes prevention programmes.

**Graphical Abstract:**

**Figure:**
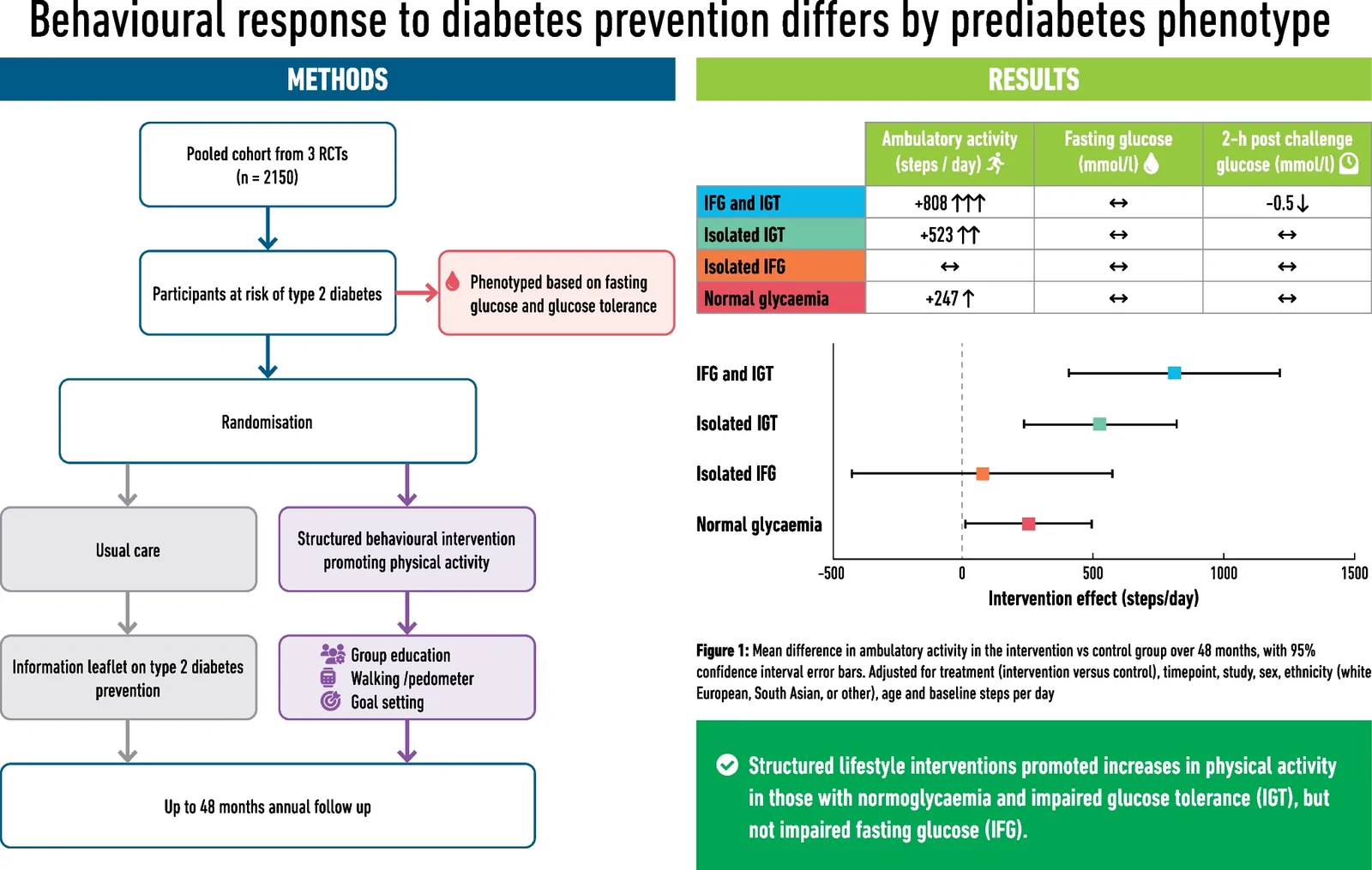

**Supplementary Information:**

The online version contains peer-reviewed but unedited supplementary material available at https://doi.org/10.1007/s00125-026-06873-5.

**Figure:**
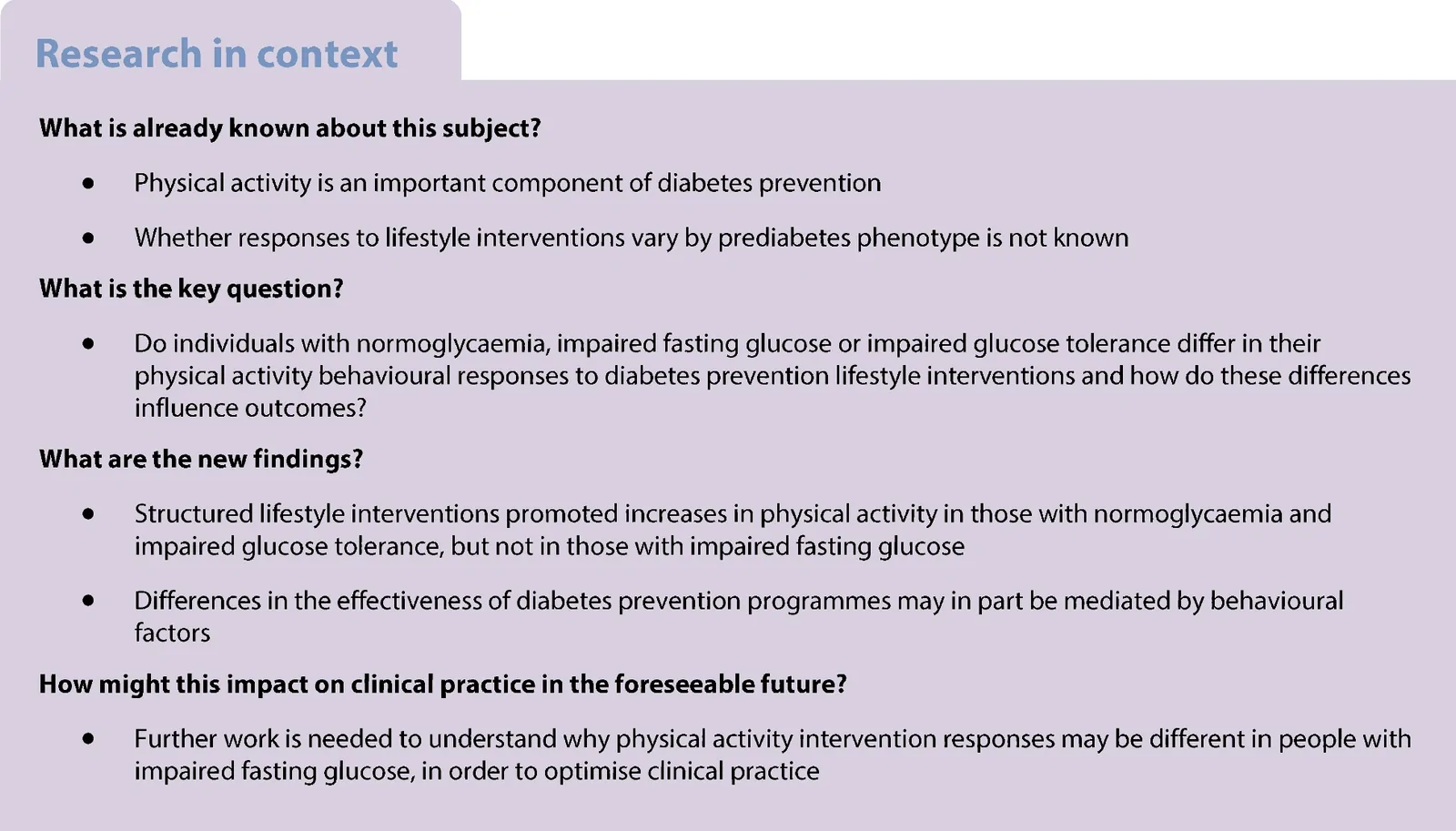

## Introduction

Prediabetes (also referred to as non-diabetic hyperglycaemia) encompasses distinct phenotypes: impaired fasting glucose (IFG; fasting glucose levels between 5.6 and 6.9 mmol/l), impaired glucose tolerance (IGT; 2 h plasma glucose levels between 7.8 and 11.0 mmol/l following oral glucose challenge), and/or HbA_1c_ levels within the prediabetes range (39–47 mmol/mol [5.7–6.4%]); these criteria identify overlapping but not identical populations [1]. The glucose-defined phenotypes (IFG and IGT), which can co-exist or occur in isolation, are often considered high-risk states for progression to type 2 diabetes, and provide important insights into underlying defects in glucose regulation [1]. An estimated 488 million and 635 million people worldwide live with IFG and IGT, respectively [2]. Prediabetes is characterised by both increased risk of deterioration to type 2 diabetes and reversibility to normoglycaemia [1, 2], providing a window of opportunity to implement preventive strategies. Targeted lifestyle interventions can successfully halt the progression of type 2 diabetes in those with prediabetes [3]. In the landmark Diabetes Prevention Programme trial in the USA, intensive lifestyle intervention reduced the incidence of type 2 diabetes by 58% over 3 years [4] and by 34% over 10 years [5], compared with the control group.

Emerging evidence shows that, when participant cohorts are stratified by prediabetes phenotype (i.e. IFG, IGT or both), the clinical response to lifestyle interventions differs [6–8]. We and others have shown using pooled data from 2794 participants (four studies) [7] and 23,684 participants (31 studies) [6] that lifestyle interventions reduced diabetes incidence and risk in people with IGT (with or without IFG), but not in those with isolated IFG. A third meta-analysis (9534 participants; 36 studies) reported that people with IGT enrolled in lifestyle interventions were more likely than those with IFG to progress to type 2 diabetes, and that glucose levels were positively altered post-intervention in the IFG group only [8]. However, in the latter study, it is unclear whether a comparison was made between intervention vs control groups or whether the results were analysed solely according to prediabetes phenotype [8].

Although there is ongoing debate regarding which prediabetes phenotype shows the greatest intervention benefit [6–8], the disparate intervention responses are probably influenced by the distinct pathophysiology of IFG and IGT. IFG is characterised by hepatic insulin resistance, normal peripheral insulin sensitivity, and an impaired early-phase insulin response to oral or intravenous glucose delivery [9, 10]. In contrast, IGT is characterised by peripheral insulin resistance and defective early- and late-phase insulin responses [9, 10]. Despite this, the tailoring of therapeutic lifestyle interventions to prediabetes sub-type is relatively underexplored [11, 12]. Furthermore, given that the success of lifestyle interventions inherently relies on participant compliance, it is important to explore whether such interventions elicit different behavioural responses across phenotypes. Physical activity is an important behavioural component of diabetes prevention, exerting positive effects on multiple tissues and organs [13], is a core component of diabetes prevention and management guidelines [14], is independently associated with diabetes prevention [15, 16], and can be objectively measured [17]. Whether people with normoglycaemia, IFG and/or IGT differ in their physical activity responses to diabetes prevention strategies is unknown. Using pooled data from three randomised controlled trials that used similar group-based lifestyle interventions for those at risk of type 2 diabetes, incorporating goal setting, pedometer use and group-based maintenance support [18–20], our objective was to examine changes in physical activity, stratified by normoglycaemia or prediabetes phenotypes.

## Methods

We pooled data from three cluster randomised controlled trials: Let’s Prevent Diabetes (Clinicaltrials.gov NCT00677937) [18, 21], Walking Away from Type 2 Diabetes (hereafter referred to as Walking Away; Clinicaltrials.gov NCT00941954) [20, 22], and PRomotion Of Physical activity through structured Education with differing Levels of ongoing Support for those with prediabetes (PROPELS; https://www.isrctn.com/ ISRCTN 83465245) [19, 23]. Randomisation was conducted at the general practitioner (GP) practice level. Participants were exposed to either usual care (control) or an intervention designed to promote increases in physical activity and other behaviours associated with diabetes risk reduction. All studies used group-based behavioural interventions, incorporating goal setting and pedometer use. The present study uses follow-up data from 12, 24 and 36 months (Let’s Prevent Diabetes and Walking Away) and from 12 and 48 months (PROPELS). The primary outcome of interest was the effect of the intervention on ambulatory activity (steps/day).

### Participants and recruitment

Age, sex and ethnicity distribution in the present pooled cohort were generally representative of the samples from which they were derived. Each study had similar inclusion criteria, and all included individuals identified as at high risk of type 2 diabetes based on their Leicester Risk Score (a composite score that uses age, self-reported sex and ethnicity, BMI, familial history of diabetes and presence of hypertension as weighted variables to determine the risk of type 2 diabetes [24]) or presence of a blood glucose or HbA_1c_ result in the prediabetes range within the last 5 years (see electronic supplementary material (ESM) Table 1). Normoglycaemia or prediabetes phenotype were defined using fasting and 2 h post-challenge glucose values collected using an oral glucose tolerance test (OGTT) performed at baseline in the original trials. The original trials were approved by research ethics committees in the Nottingham National Health Service (NHS) (Walking Away) or NHS East Midlands (Let’s Prevent Diabetes and PROPELS). All participants provided written informed consent for the original research and for the storage and use of samples for future research.

### Intervention

#### Walking Away

GP practices were randomised (1:1) to receive usual care (control) or the Walking Away intervention [20, 22]. Participants assigned to the intervention attended a 3 h group-based structured education programme with the primary aim of promoting an increase in physical activity. Progress review and behaviour change maintenance support were provided via 2 h follow-on group-based sessions at 12 and 24 months, and by 15 min telephone calls at 6, 18 and 30 months.

#### PROPELS

Participants were randomised (1:1:1) to usual care, the Walking Away group or the Walking Away Plus group [19, 23]. The Walking Away programme was delivered as described above, including a group-based structured education programme, progress reviews and behaviour change maintenance support via 2 h follow-on group-based sessions at 12 and 24 months, and by 15 min telephone calls at 6, 18 and 30 months. The Walking Away Plus group also received an additional bespoke text-messaging and telephone call follow-on service. The Walking Away and Walking Away Plus groups were pooled into a single intervention group for the purpose of the present analysis.

#### Let’s Prevent Diabetes

GP practices were randomised (1:1) to receive usual care (control) or the Let’s Prevent Diabetes intervention [18, 21], which was based on Walking Away but with an additional focus on weight regulation and nutrition, comprising a 6 h group-based structured education programme, 3 h refresher sessions at 12 and 24 months, and a 15 min telephone call every 3 months to facilitate progress review and behaviour change maintenance.

### Common approach to physical activity promotion

The approach to physical activity promotion in all three trials was based on the content and behaviour change techniques of the successful Prediabetes Risk Education and Physical Activity Recommendation and Encouragement (PREPARE) programme; a full description of the rationale, development and efficacy of which can be found elsewhere [25, 26]. In all trials, interventions were based on group-based behavioural theory-driven interventions with up to ten participants per group, aimed at increasing knowledge of diabetes and diabetes prevention alongside promoting health behaviour change, including increased physical activity. This included the use of self-regulatory strategies, which for physical activity were centred on steps per day goal setting and self-monitoring through the provision of pedometers. Goals were typically based on increasing physical activity levels by 3000 steps/day but were personalised to individual ability and preference. Goal attainment was supported using proximal objectives, such as increasing steps by 500 per day every 2 weeks. The minimum clinically important difference in ambulatory activity is 500 steps per day for inactive adults [27].

### Usual care

Control participants in each study received usual care plus a standardised information booklet on type 2 diabetes risk and prevention. Based on Leventhal’s common-sense model [28], the leaflet used five domains (causes, consequences, identity, control/treatment and timeline) to address the impact of physical activity and lifestyle change on the onset and progression of type 2 diabetes.

### Physical activity measurement

Steps per day were measured using sealed NL-800 piezoelectric pedometers (New Lifestyles, Lee’s Summit, MO, USA) (Let’s Prevent Diabetes) or ActiGraph GT3X accelerometers (ActiGraph, Pensacola, FL, USA) (Walking Away and PROPELS). Pedometers or accelerometers were placed on the trunk along the right anterior axillary line or on the right mid-axillary line at the hip, and were worn during waking hours for seven consecutive days. At least three valid days of data were required for the present analysis; a valid day constituted at least 10 h of wear time. Data from each measurement device were pooled at the individual level as reported in a previous analysis [29]. Both types of device use piezoelectric technology to measure physical activity, which shows a high level of accuracy when measuring steps taken [30]; accordingly, these trials are considered comparable in their assessments of steps per day.

### Participant characteristics

This study reports data on various demographic characteristics collected in the original trials [18–20]: age, sex, ethnicity and BMI. All variables were captured using the same standard operating procedures. Fasting and 2 h post-challenge glucose values were assessed using OGTT at each clinical measurement session, after a 12 h fast and 24 h of avoiding vigorous-intensity exercise. For the PROPELS study, OGTT data were collected from participants at the Leicester site only. Intervention effects on anthropometric variables, glucose variables and other outcomes of interest have been published previously elsewhere [18–20].

### Statistical analyses

Demographic characteristics are reported as median (IQR) or *n* (%). The results for pooled physical activity, fasting glucose and 2 h post-challenge glucose over time and their intervention effects are reported as means ± SD and means (95% CI).

Analyses were conducted on pooled individual level data. The intervention effect was calculated using generalised estimating equations with an exchangeable matrix taking into account repeated follow-up measures over time (12, 24, 36 and 48 month follow-up) and allowing for clustering by GP practice. Steps per day at follow-up was the dependent variable. Models were a priori stratified by normoglycaemia or prediabetes phenotype, and adjusted for treatment (intervention vs control), timepoint, trial, sex, ethnicity (White European, South Asian, or other), age and baseline steps per day. Treatment × time interaction terms were used to assess whether the intervention effect varied by time (across 12, 24, 36 and 48 months). For sensitivity analyses, additional models were stratified by BMI (<30 kg/m^2^ and ≥30 kg/m^2^), sex and study. *p* values for the treatment × glucose status interaction terms were obtained from a pooled generalised estimating equation model adjusted as before. Additionally, change in physical activity over time was regressed against the phenotypes to determine difference in behavioural change irrespective of group allocation.

Generalised estimating equation models assessed the change in 2 h post-challenge glucose (mmol/l) and fasting glucose (mmol/l) over the four follow-up timepoints, adjusted for their respective baseline values plus treatment, timepoint, trial, sex, ethnicity and age. To investigate whether intervention effects on fasting or post-challenge glucose were independent of physical activity, significant effects were further adjusted for baseline and change in ambulatory activity.

Data were analysed using SPSS Statistics software version 28 (IBM). A *p* value <0.05 was considered significant for main effects and interactions.

## Results

**Fig. 1:**
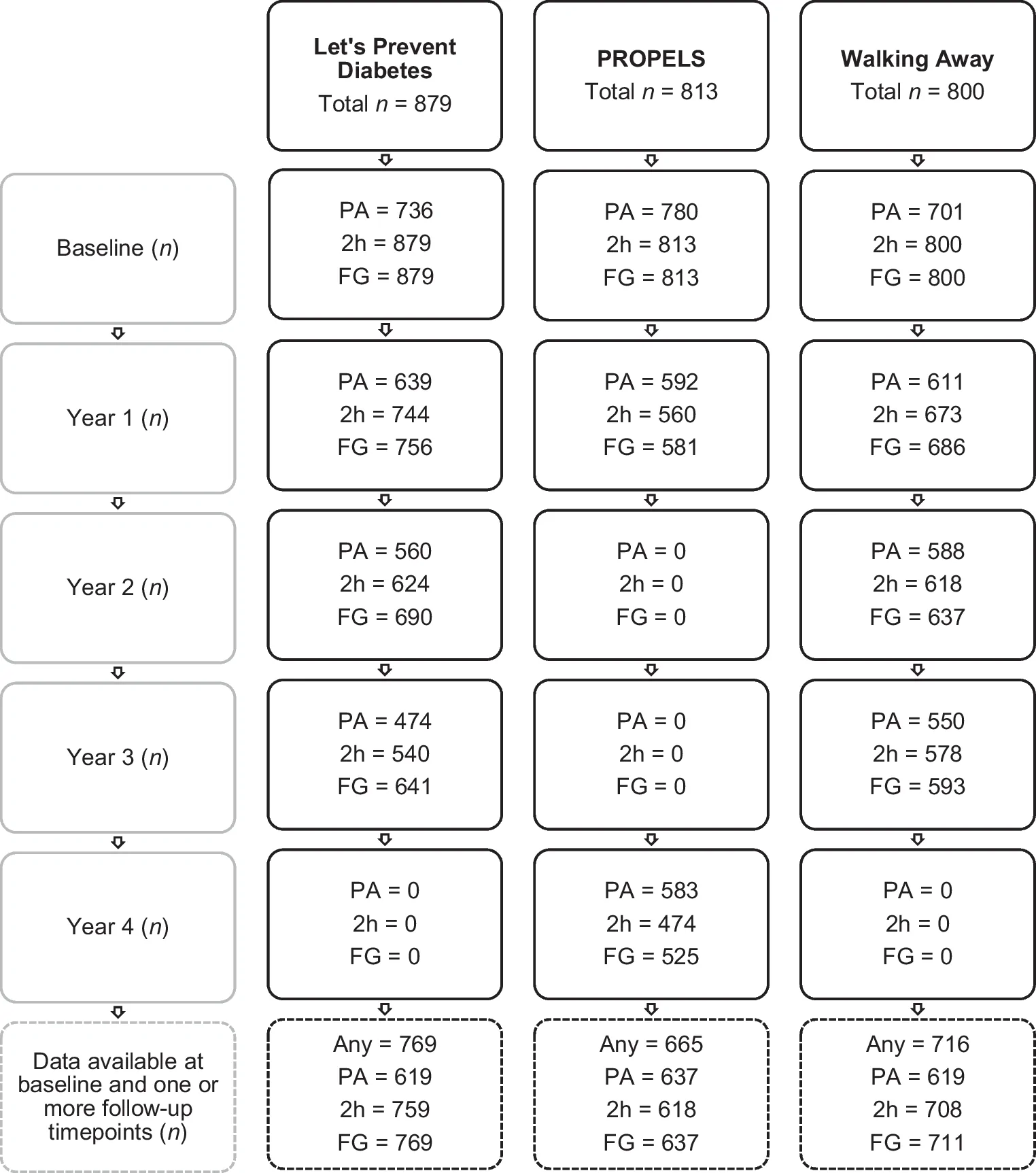
CONSORT diagram of participants with valid data for physical activity (PA), 2 h post-challenge glucose levels (2h) and fasting glucose levels (FG) at each timepoint, stratified by study. The flow diagram depicts participant selection for the present pooled post hoc analysis. Withdrawals and losses to follow-up within the individual randomised controlled trials are reported in the original trial publications and are not shown here. Exclusions shown in the diagram relate only to the availability of data required for the current analysis

In the original trials, the number of participants totalled 879, 813 and 800 (Leicester site only) for Let’s Prevent Diabetes, PROPELS and Walking Away, respectively. Of these, ambulatory activity data were available at baseline and at least one follow-up timepoint for 619, 637 and 619 participants for the Let’s Prevent Diabetes, PROPELS and Walking Away trials, respectively. Equivalent data availability for 2 h post-challenge glucose and fasting glucose were 759, 618 and 708, respectively, and 769, 637 and 711, respectively. A total of 2150 participants had complete data (baseline and at least one follow-up timepoint) for one or more outcome of interest (Fig. 1 and ESM Fig. 1). Demographic characteristics are reported in Table 1. The mean baseline steps per day was highest in the participants with isolated IFG (7244 ± 3489), compared to participants with normoglycaemia (6130 ± 3201), isolated IGT (5984 ± 2904) or IFG and IGT (6310 ± 2872). Values for the intervention and control groups separately are shown in Table 2.

**Table 1:** Demographic characteristics

	Normoglycaemia		Isolated IFG		Isolated IGT		IFG and IGT	
	Control (*n*=382)	Intervention (*n*=446)	Control (*n*=122)	Intervention (*n*=151)	Control (*n*=330)	Intervention (*n*=357)	Control (*n*=155)	Intervention (*n*=207)
Age (years)	63 (56–68)	62 (55–67)	63 (54–69)	63 (57–66)	65 (60–71)	65 (60–69)	65 (58–69)	64 (58–69)
Men	217 (56.8)	250 (56.1)	91 (74.6)	100 (66.2)	192 (58.2)	223 (62.5)	100 (64.5)	127 (61.4)
BMI (kg/m^2^)	30.4 (27.2–34.4)	29.7 (26.5–33.4)	30.4 (27.7–36.2)	29.4 (26.7–33.0)	31.7 (28.6–35.5)	30.3 (27.4–34.2)	31.2 (28.7–35.9)	31.9 (28.3–35.1)
Ethnicity								
White European	305 (79.8)	330 (74.0)	100 (82.0)	116 (76.8)	262 (79.4)	291 (81.5)	130 (83.9)	169 (81.6)
South Asian	41 (10.7)	65 (14.6)	14 (11.5)	22 (14.6)	12 (3.6)	10 (2.8)	13 (8.4)	18 (8.7)
Other^a^	36 (9.4)	51 (11.4)	8 (6.6)	13 (8.6)	56 (17.0)	56 (15.7)	12 (7.7)	20 (9.7)
Study cohort								
PROPELS	116 (30.4)	197 (44.2)	54 (44.3)	80 (53.0)	27 (8.2)	61 (17.1)	48 (31.0)	82 (39.6)
Walking Away	266 (69.6)	249 (55.8)	23 (18.9)	24 (15.9)	50 (15.2)	57 (16.0)	16 (10.3)	31 (15.0)
Let’s Prevent Diabetes	0 (0.0)	0 (0.0)	45 (36.9)	47 (31.1)	253 (76.7)	239 (66.9)	91 (58.7)	94 (45.4)

Values for continuous variables are median (IQR); those for categorical variables are *n* (%)

Baseline characteristics are presented for participants with complete data (baseline and one or more follow-up timepoint) for physical activity, fasting glucose or 2 h post-challenge glucose levels

^a^‘Other’ includes any other White background, mixed background, Black or Black British, Chinese and any other Asian background

**Table 2:** Pooled physical activity, fasting glucose and 2 h post-challenge glucose in the control and intervention groups over time, and associated intervention effect

	Normoglycaemia			Isolated IFG			Isolated IGT			IFG and IGT		
Timepoint (years)	*n*	Control	Intervention	*n*	Control	Intervention	*n*	Control	Intervention	*n*	Control	Intervention
Ambulatory activity (steps/day)												
Baseline	745	5836 ± 3075	6378 ± 3287	240	7201 ± 3449	7279 ± 3534	580	6029 ± 2985	5942 ± 2832	310	6330 ± 2951	6294 ± 2818
1	712	6582 ± 3147	7287 ± 3067	230	7047 ± 3636	7527 ± 3214	590	5933 ± 2837	6466 ± 2782	307	5766 ± 2695	6809 ± 3402
2	431	6259 ± 2813	6529 ± 3142	98	6492 ± 3552	6956 ± 3725	450	5768 ± 2803	6469 ± 3420	167	6099 ± 2794	6425 ± 3353
3	411	6166 ± 2827	6580 ± 3192	91	6515 ± 3358	6226 ± 3645	377	5283 ± 2860	5892 ± 2884	144	5772 ± 2693	5812 ± 2870
4	271	6265 ± 3045	6725 ± 3101	117	7224 ± 2905	7482 ± 3145	81	5224 ± 1950	6230 ± 3246	114	5409 ± 2929	6229 ± 3544
Intervention effect	745		247 (4, 491)	240		71 (−429, 571)	580		523 (229, 818)	310		808 (402, 1213)
*p* value			0.047			0.781			<0.001			<0.001
2 h post-challenge glucose (mmol/l)												
Baseline	801	5.4 ± 1.3	5.5 ± 1.2	262	6.0 ± 1.2	6.1 ± 1.1	675	9.0 ± 1.2	9.1 ± 1.1	347	9.8 ± 1.6	9.9 ± 1.6
1	741	5.2 ± 1.5	5.4 ± 1.6	247	6.4 ± 2.2	6.4 ± 2.0	652	7.4 ± 2.0	7.7 ± 2.2	335	8.8 ± 2.5	8.7 ± 2.6
2	443	5.3 ± 1.7	5.4 ± 1.8	116	6.3 ± 2.0	6.6 ± 2.0	512	7.5 ± 2.1	7.7 ± 2.3	170	8.9 ± 2.3	8.3 ± 2.2
3	433	5.4 ± 1.5	5.5 ± 1.7	99	6.3 ± 1.9	7.0 ± 2.9	456	7.6 ± 2.2	7.7 ± 2.6	129	8.3 ± 2.4	8.1 ± 2.2
4	230	6.4 ± 2.1	6.5 ± 2.0	97	7.4 ± 2.3	7.1 ± 2.1	62	9.0 ± 2.6	8.4 ± 2.4	85	11.2 ± 4.0	9.1 ± 3.8
Intervention effect	801		0.1 (−0.1, 0.3)	262		0.0 (−0.4, 0.5)	675		0.1 (−0.2, 0.4)	347		−0.5 (−0.9, −0.0)
*p* value			0.308			0.857			0.477			0.039
Fasting glucose (mmol/l)												
Baseline	810	5.1 ± 0.4	5.2 ± 0.4	268	6.4 ± 0.3	6.4 ± 0.4	683	5.3 ± 0.5	5.3 ± 0.4	356	6.4 ± 0.4	6.5 ± 0.5
1	764	5.2 ± 0.6	5.3 ± 0.7	250	6.2 ± 0.7	6.2 ± 0.7	662	5.4 ± 0.6	5.4 ± 0.7	345	6.3 ± 0.8	6.4 ± 0.9
2	461	5.1 ± 0.5	5.1 ± 0.5	122	6.2 ± 0.6	6.0 ± 0.7	545	5.4 ± 0.7	5.4 ± 0.7	197	6.2 ± 0.7	6.2 ± 0.8
3	447	5.1 ± 0.5	5.1 ± 0.5	111	6.1 ± 0.7	6.3 ± 1.3	501	5.5 ± 0.7	5.4 ± 0.7	173	6.1 ± 0.7	6.2 ± 0.8
4	245	5.9 ± 0.9	5.9 ± 0.8	110	6.6 ± 1.2	6.7 ± 1.0	69	5.9 ± 0.8	6.3 ± 1.1	101	7.5 ± 1.2	7.0 ± 1.3
Intervention effect	810		−0.0 (−0.1, 0.0)	268		−0.0 (−0.2, 0.1)	683		0.0 (−0.1, 0.1)	356		−0.1 (−0.2, 0.1)
*p* value			0.419			0.567			0.690			0.363

Data for variables are means ± SD; values for the intervention effect are mean (95% CI). Intervention effects were calculated using generalised estimating equations with an exchangeable matrix, and display the mean difference in the intervention group relative to the control group during follow-up, adjusted for age, sex, ethnicity, study, baseline values and follow-up timepoint

### Normoglycaemia

**Fig. 2:**
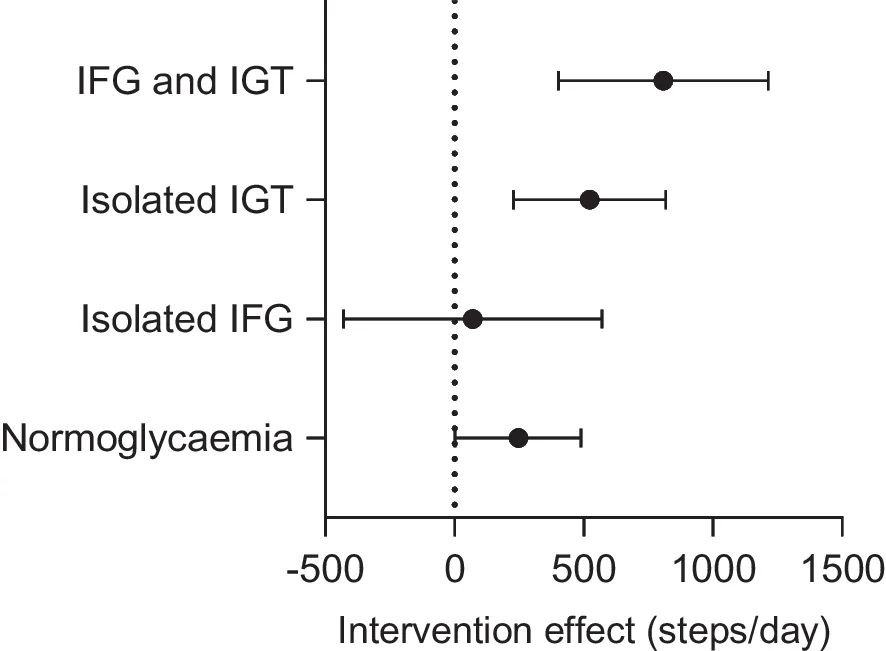
Mean differences in ambulatory activity in the intervention vs control groups over 48 months, with 95% CI error bars. Adjusted for treatment (intervention vs control), timepoint, study, sex, ethnicity (White European, South Asian, or other), age and baseline steps per day

Of the participants with complete data (at baseline and at least one follow-up timepoint for one or more outcome of interest), 828 (38.5%) were in the normoglycaemia group. Ambulatory activity increased over 48 months in the intervention groups relative to usual care (mean 247 steps/day; 95% CI 4, 491; *p*=0.047) (Fig. 2 and Table 2). There was no change in 2 h post-challenge glucose levels (mean 0.1 mmol/l; 95% CI −0.1, 0.3; *p*=0.308) or fasting glucose levels (mean −0.0 mmol/l; 95% CI −0.1, 0.0; *p*=0.419) over 48 months (Table 2).

### IFG

Of the participants with complete data, as described above, 273 (12.7%) had IFG. There was no change in ambulatory activity (mean 71 steps/day; 95% CI −429, 571; *p*=0.781), 2 h post-challenge glucose levels (mean 0.0 mmol/l; 95% CI −0.4, 0.5; *p*=0.857) or fasting glucose levels (mean −0.0 mmol/l; 95% CI −0.2, 0.1; *p*=0.567) over 48 months (Fig. 2 and Table 2).

### IGT

Of the participants with complete data, as described above, 687 (32.0%) had IGT. Ambulatory activity increased over 48 months in the intervention groups relative to usual care (mean 523 steps/day; 95% CI 229, 818; *p*<0.001) (Fig. 2 and Table 2). There was no change in 2 h post-challenge glucose levels (mean 0.1 mmol/l; 95% CI −0.2, 0.4; *p*=0.477) or fasting glucose levels (mean 0.0 mmol/l; 95% CI −0.1, 0.1; *p*=0.690) over 48 months (Table 2).

### IFG and IGT

Of the participants with complete data, as described above, 362 (16.8%) had IFG and IGT. Ambulatory activity increased over 48 months in the intervention groups relative to usual care (mean 808 steps/day; 95% CI 402, 1213; *p*<0.001) (Fig. 2 and Table 2). There was an effect for 2 h post-challenge glucose levels (mean −0.5 mmol/l; 95% CI −0.9, −0.0; *p*=0.039) but not fasting glucose (mean −0.1 mmol/l; 95% CI −0.2, 0.1; *p*=0.363) (Table 2). The effect for 2 h post-challenge glucose was attenuated after further adjustment for baseline and change in physical activity (mean −0.3 mmol/l; 95% CI −0.9, 0.1; *p*=0.131).

### Interaction effect

The intervention effect for physical activity differed by phenotype. The effect in the isolated IFG group was different from that in the isolated IGT group (*p*=0.017) and combined IFG and IGT group (*p*=0.021). The effect in the isolated IFG group was not different from that in the normoglycaemia group (*p*=0.793).

### Sensitivity analyses

Physical activity effects were consistent across study, sex and BMI (ESM Tables 2, 3 and 4, respectively), excluding in those with isolated IGT where the intervention effect was greater in men than in women (*p*=0.003). When regressed against the phenotypes, the change in physical activity over time was similar across groups (ESM Table 5).

## Discussion

Using pooled data from three randomised controlled trials, we examined behavioural responses to lifestyle interventions aimed at diabetes prevention across glucose-defined prediabetes phenotypes. Over a follow-up period of up to 48 months, ambulatory activity (steps per day) increased in people with normoglycaemia, isolated IGT and IFG+IGT, but not in those with isolated IFG, relative to control conditions. The difference in change in steps per day between the intervention and control groups reached the threshold for the minimum clinically important difference (500 steps/day) in those with isolated IGT and in those with both IFG and IGT [27].

A recent systematic review identified baseline dysglycaemia as one of the few characteristics for which there is evidence of an effect on diabetes prevention intervention effectiveness, although the certainty of the evidence was low [31]. Our findings extend this evidence by suggesting that heterogeneity within prediabetes phenotypes may be associated with differential physical activity responses to intervention, providing a potential reason for variation in clinical outcomes. The cohort with both IFG and IGT demonstrated the most favourable behavioural response to the intervention, with the highest increase in steps per day of all subgroups (Table 2). It should be noted that differences between the control and intervention groups for those with IGT were driven by decreasing levels of physical activity over time in the control group, with maintenance of activity levels in the intervention group. However, against the backdrop of faster functional decline in individuals with prediabetes compared to those with normoglycaemia [32], maintenance of physical activity over time in an ageing high-risk population is clinically important.

The increase in physical activity in those with IFG and IGT was associated with the most favourable clinical response, being the only subgroup to elicit a reduction in 2 h post-challenge glucose levels (mean −0.5 mmol/l; 95% CI −0.9, −0.0), consistent with an estimated 4% lower mortality risk [33]. A smaller, but clinically meaningful [27], increase in physical activity was observed in those with isolated IGT, although this did not appear sufficient to alter 2 h post-challenge or fasting glucose levels. The increase in physical activity in the normoglycaemia group did not reach the threshold for clinical meaningfulness.

We observed no significant change in physical activity following intervention in the IFG subgroup. Clinical non-response to diabetes prevention strategies (defined as either failure to regress to normal glucose regulation or progression to type 2 diabetes) is commonly reported in individuals with isolated IFG [6, 7]. In agreement, we found no intervention effect on fasting plasma glucose or 2 h post-challenge glucose in this subgroup. The disparate intervention response in comparison to individuals with IGT, who often show improved prognosis following intervention, has previously been attributed to the distinct pathophysiology of IFG and IGT [6, 7]. Our findings suggest an additional, and potentially complementary, explanation: differences in the physical activity response to intervention may contribute to the differential clinical benefits observed across glycaemic phenotypes. The potential for behaviour responses to partly mediate clinical responses is supported by the attenuation of 2 h post-challenge glucose level reductions in those with IFG and IGT after adjustment for change in physical activity (mean −0.3 mmol/l; 95% CI −0.9, 0.1). Accordingly, it may be speculated that the greater clinical responses to lifestyle interventions in the highest risk IGT groups are partly driven by greater behavioural responses, despite being given comparable interventions. The largely consistent intervention effects in women and men suggest that the findings are broadly generalisable across sex. However, among participants with isolated IGT, the intervention effect was greater in men than in women, suggesting possible sex-related heterogeneity of treatment response in this subgroup.

Several studies have investigated barriers to physical activity in people with prediabetes, identifying stress, lack of time and motivation, feeling incompetent and extrinsic social determinants of health as influencing factors [34, 35]. However, these studies did not differentiate between prediabetes phenotypes. It is possible that differences in physical activity responses could be driven, at least in part, by differences in demographic characteristics and lifestyle behaviours. A greater proportion of those with IFG were men [36]. Furthermore, the current study shows that those with IFG have the highest levels of physical activity at baseline (>7000 steps/day), highlighting a potential ceiling effect, whereby higher levels of pre-existing habitual physical activity may limit opportunity for increasing physical activity further. However, the specific reasons for the poor physical activity response to lifestyle interventions in those with isolated IFG remain to be elucidated.

Our findings support the importance of precise phenotyping of people with prediabetes in clinical practice to optimise proactive and personalised standard care. Strengths of this study include the objective assessment of physical activity and oral glucose tolerance in 2150 adults at risk of type 2 diabetes who took part in randomised controlled trials with long-term follow-up. We used standardised outcomes and interventions. A potential limitation is the pooling of three large datasets. Some methodological differences existed between the interventions, including follow-up timepoints and the duration of the core group-based education session (6 h in Let’s Prevent Diabetes and 3 h in Walking Away and PROPELS). However, each study followed the same standard operating procedures for data collection, models were adjusted for differences between datasets, and sensitivity analyses showed that results were consistent across studies. Finally, this is a post hoc analysis of studies that were not originally designed to address this research question, and the results should be interpreted accordingly.

### Conclusions

Structured behavioural interventions promoted clinically meaningful increases in physical activity in those with IGT compared with those receiving usual care, but not in those with IFG. Previously observed differences in the effectiveness of diabetes prevention programmes may in part be mediated by a differential physical activity response to the intervention among glycaemic phenotypes. These findings suggest that consideration of glycaemic phenotype may help to identify individuals who are less likely to respond to standard lifestyle interventions, supporting the development of more targeted and personalised diabetes prevention strategies. Optimising type 2 diabetes prevention is of clinical importance, and further work is needed to understand why the behavioural response to lifestyle interventions is lower in people with IFG.

## Supplementary Information

Below is the link to the electronic supplementary material.

ESM (PDF 336 KB)

## Data Availability

De-identified individual participant data that underlie the results reported in this article will be made available upon reasonable request to the corresponding author after publication. Requests will be reviewed by the chief investigator. Access will be provided following approval of a methodologically sound proposal and completion of an appropriate data sharing agreement, in accordance with participant consent and UK data protection regulations.

## Abbreviations

GP: General practitioner
IFG: Impaired fasting glucose
IGT: Impaired glucose tolerance
