# Supplementary material for: Differential physical activity responses to diabetes risk reduction interventions according to prediabetes phenotype in at-risk individuals: pooled analysis from three randomised controlled trials
Source: Diabetologia. 2026 Sep 14;69(12):3548–58. doi: 10.1007/s00125-026-06873-5 (PMC13714053; doi:10.1007/s00125-026-06873-5)
Supplement: Supplementary file 1 [file 125_2026_6873_MOESM1_ESM.pdf]

Electronic Supplementary Material

ESM Table 1 Participant inclusion criteria and recruitment.

	Walking Away	Let's Prevent Diabetes	PROPELS
Identification of participants	GP databases within Leicestershire, UK.	GP databases within Leicestershire, UK.	GP databases within Leicestershire and Cambridge, UK. Research databases within Cambridge, UK.
**Inclusion criteria**			
Age	18-74 years.	40–74 years if white European, or aged 25–74 years if South Asian.	40–74 years if white European, or aged 25–74 years if South Asian.
Sex	Men and women	Men and women	Men and women
Risk of type 2 diabetes	Scoring within the 90th percentile of a modified version of the automated Leicester Risk Score, when individuals were ranked for diabetes risk using predefined weighted variables [1].	Scoring within the 90th percentile of a modified version of the automated Leicester Risk Score, when individuals were ranked for diabetes risk using predefined weighted variables [1].	Blood glucose or HbA1c result recorded in the prediabetes range within the last 5 years.

Reference

1. Gray LJ, Taub NA, Khunti K, Gardiner E, Hiles S, Webb DR, Srinivasan BT, Davies MJ. The Leicester Risk Assessment score for detecting undiagnosed Type 2 diabetes and impaired glucose regulation for use in a multiethnic UK setting. Diabet Med. 2010 Aug;27(8):887-95. doi: 10.1111/j.1464-5491.2010.03037.x. PMID: 20653746.

ESM Table 2 Intervention effect for pooled physical activity in the intervention versus control group stratified by study.

	Ambulatory activity (steps/day)	Interaction *p*-value
**Normal glycaemia**		
Walking Away	239 (-67, 545)	0.904
Let's Prevent Diabetes	N/A	
PROPELS	220 (-192, 633)	
**Isolated IFG**		
Walking Away	135 (-1217, 1488)	0.427
Let's Prevent Diabetes	-97 (-1112, 918)	
PROPELS	352 (-184, 888)	
**Isolated IGT**		
Walking Away	317 (229, 863)	0.712
Let's Prevent Diabetes	617 (238, 995)	
PROPELS	655 (28, 1283)	
**IFG and IGT**		
Walking Away	655 (-362, 1673)	0.649
Let's Prevent Diabetes	674 (50, 1298)	
PROPELS	971 (353, 1588)	

Data as mean (95% confidence interval). Intervention effects were calculated using generalised estimating equations with an exchangeable matrix and display the average difference in the intervention group relative to the control group during follow-up, adjusted for age, sex, ethnicity, baseline values, and follow-up time point.

ESM Table 3 Intervention effect for pooled physical activity in the intervention versus control group stratified by sex.

	Ambulatory activity (steps/day)	Interaction p-value
**Normal glycaemia**		
Men	280 (-80, 641)	0.984
Women	205 (-98, 508)	
**Isolated IFG**		
Men	33 (-593, 658)	0.840
Women	152 (-566, 870)	
**Isolated IGT**		
Men	897 (487, 1307)	0.003
Women	21 (-378. 421)	
**IFG and IGT**		
Men	681 (130, 1232)	0.394
Women	1143 (592, 1694)	

Data as mean (95% confidence interval). Intervention effects were calculated using generalised estimating equations with an exchangeable matrix and display the average difference in the intervention group relative to the control group during follow-up, adjusted for age, ethnicity, study, baseline values, and follow-up time point.

ESM Table 4 Intervention effect for pooled physical activity in the intervention versus control group stratified by BMI.

	Ambulatory activity (steps/day)	Interaction *p*-value
**Normal glycaemia**		
<30 kg/m^2	360 (9, 711)	0.284
≥30 kg/m^2	147 (-177, 472)	
**Isolated IFG**		
<30 kg/m^2	208 (-402, 818)	0.554
≥30 kg/m^2	-59 (-851, 733)	
**Isolated IGT**		
<30 kg/m^2	471 (24, 917)	0.800
≥30 kg/m^2	488 (85, 890)	
**IFG and IGT**		
<30 kg/m^2	908 (249, 1566)	0.727
≥30 kg/m^2	833 (292, 1374)	

Data as mean (95% confidence interval). Intervention effects were calculated using generalised estimating equations with an exchangeable matrix and display the average difference in the intervention group relative to the control group during follow-up, adjusted for age, sex, ethnicity, study, baseline values, and follow-up time point.

ESM Table 5 Change in ambulatory activity across phenotypes.

	Ambulatory activity (steps/day)
**Normal glycaemia**	126 (-55, 306)
**Isolated IFG**	116 (-164, 397)
**Isolated IGT**	-157 (-346, 31)
**IFG and IGT**	-111 (-349, 126)

Data as mean (95% confidence interval). Effects were calculated using generalised estimating equations with an exchangeable matrix and display the average change in ambulatory activity during follow-up, adjusted for age, sex, group, phenotype, ethnicity, study, baseline values, and follow-up time point.

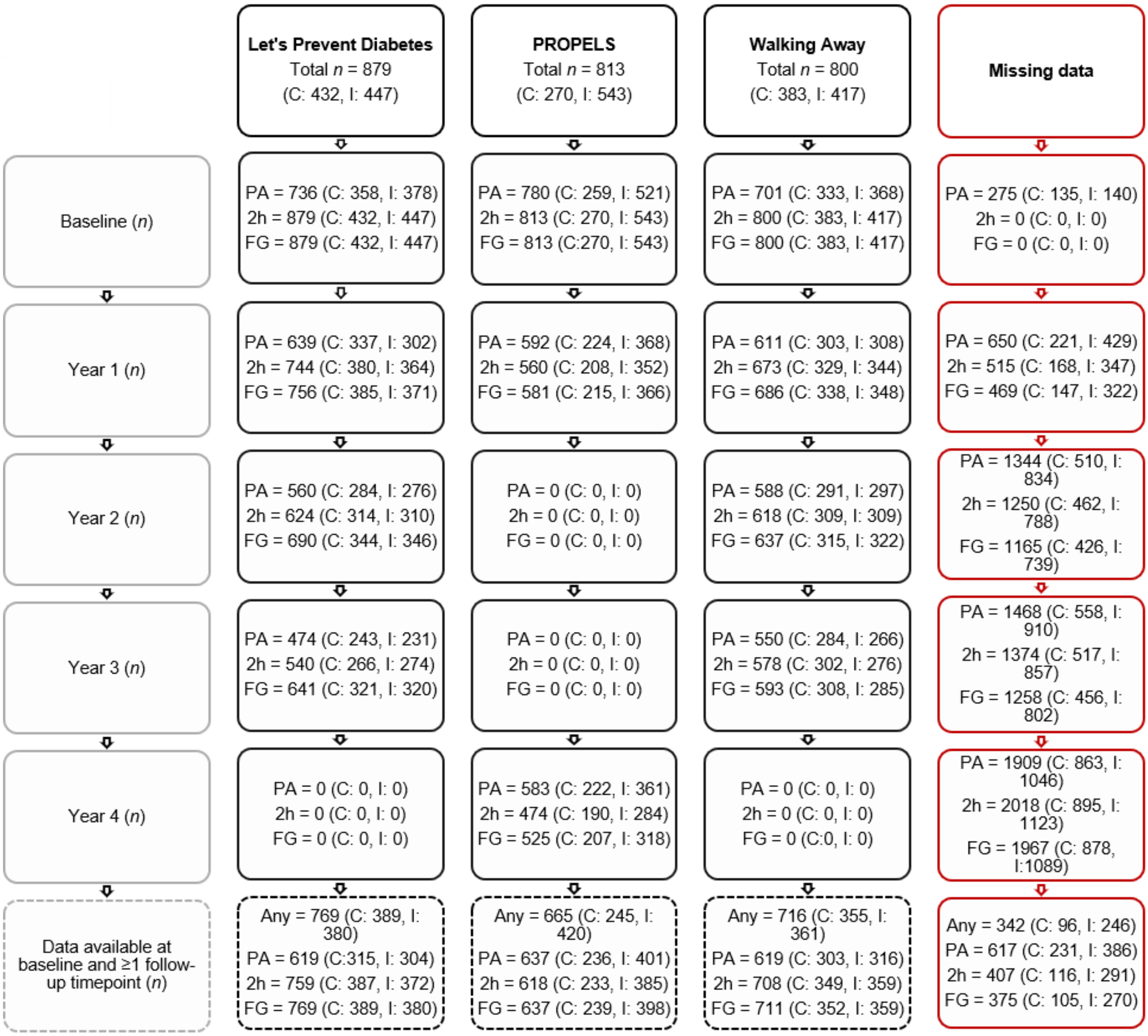

ESM Fig. 1 Consort diagram of participants with valid and missing data for physical activity (PA), 2-hour post-challenge glucose (2h) and fasting glucose (FG) levels in the intervention (I) and control (C) groups at each time point, stratified by study. The flow diagram depicts participant selection for the present pooled post-hoc analysis. Withdrawals and losses to follow-up within the individual randomised controlled trials are reported in the original trial publications and are not shown here. Exclusions shown in the diagram relate only to the availability of data required for the current analysis.
